# Variability in the Response against *Teladorsagia circumcincta* in Lambs of Two Canarian Sheep Breeds

**DOI:** 10.3390/ijms24010029

**Published:** 2022-12-20

**Authors:** Tara Pérez-Hernández, Julia N. Hernández, Cynthia Machín, Tom N. McNeilly, Alasdair J. Nisbet, Jacqueline B. Matthews, Stewart T. G. Burgess, Jorge F. González

**Affiliations:** 1 Instituto Universitario Sanidad Animal y Seguridad Alimentaria, Facultad de Veterinaria, Universidad de Las Palmas de Gran Canaria, 35413 Arucas, Spain; 2 Moredun Research Institute, Edinburgh EH26 0PZ, UK; 3 Austin Davis Biologics, Ltd., Northamptonshire NN14 4BL, UK

**Keywords:** gastrointestinal nematodes, genetic resistance, sheep, immunology, transcriptomic analysis

## Abstract

The increasing resistance to anthelmintics has necessitated the exploration of alternative control strategies of gastrointestinal nematode (GIN) infections. A sustainable option is genetic selection based on differences in susceptibility to GIN infection between and within breeds of sheep. Here, three-month-old Canaria Hair breed (GIN-resistant) and Canaria Sheep breed (GIN-susceptible) showed no significant between-breed differences after trickle infection with *Teladorsagia circumcincta*, whereas considerable individual variability was found in both breeds. Next, data from lambs of both breeds were used to explore the relationships between parasitological variables and *T. circumcincta*-specific IgA levels, local immune cell populations, and abomasal lymph node gene expression to understand the possible mechanisms underlying resistance. Mucosal IgA levels as well as numbers of globular leukocytes and MHC-II^+^ cells were associated with protection. Analysis of lymph node gene expression revealed the associations between lower parasite numbers and cumulative fecal egg counts and several immune pathways, such as leukocyte cell adhesion, activation and differentiation of T cells, in particular CD4^+^ and IL-4 production. The data obtained here may inform on the relationship between phenotypic resistance variability and protective responses at the humoral, cellular, and transcriptomic levels, thus contributing to identifying immune responses in young lambs that could be used as markers for selection.

## 1. Introduction

Gastrointestinal nematodes (GIN) represent an increasing concern for the domesticated ruminant farming sector. Recent estimates of direct and indirect losses attributable to GIN in ruminants amount to approximately EUR 686 million per year in Europe alone [1]. Despite resistance against the main anthelmintic classes being reported worldwide [2,3,4,5,6], the farming sector still relies largely on chemotherapeutic intervention to control ovine GIN. This, alongside the climate-driven expansion of GIN prevalence across the globe, poses a serious threat to the economic and environmental sustainability of the sector [7]. Consequently, the quest for alternative approaches to management based on more restricted use of anthelmintics, such as pasture management, nutritional supplementation, biological control, vaccine development, or genetic selection has increased in importance [8].

Genetic selection relies on purposely breeding individuals with the ability to prevent worm establishment and/or to expel parasitic nematodes based on the timely development of an effective immune response [9]. This usually manifests as reductions in egg excretion, worm burden, and fecundity after the development of a type 2 immune response, characterised by CD4 activation, increases in Th2 (IL-4, IL-13 and IL-5) cytokine levels, IgE and IgA production and recruitment of mast cells, globule leukocytes, and eosinophils in parasitised tissues [10]. However, there is a lack of comprehensive data on how these immune response mechanisms operate in young lambs. It has been suggested that lambs generally need repeated exposure during their first year to fully acquire protective immunity, even though some individuals demonstrate repeatable low GIN egg excretion from three months of age and demonstrate some features of an effective immune response [11,12]. In addition, some sheep breeds, such as the Canaria Hair Breed (CHB), display a natural resistance against GIN, with evidence that such breeds could manifest resistance mechanisms at a younger age than other breeds [13,14,15,16,17,18]. In this regard, an in-depth study of the phenotypic variation in lambs within and between breeds, and the genetic basis of this variation, could improve understanding of the immunological dynamics that participate in resistance against nematodes in young lambs. This is important as many of the key impacts of ovine GIN infection on production occur in early life. To this end, global transcriptomic analyses have been used to explore changes in gene expression in host tissue throughout infection and to characterise the immune-related pathways associated with the resistance across breeds in an unbiased manner [19,20,21]. *Teladorsagia circumcincta* was used in this study as previous data have described that this is one of the most prevalent species in small ruminants in the Canary Islands, along with *Haemonchus contortus* and *Trichostrongylus* spp., with prevalence reaching around 65.8% in some areas of this territory [22].

The present study aimed to determine whether there are breed and/or individual differences in the resistance in three-month-old lambs of either worm-resistant (CHB) or susceptible (Canaria breed, CS) breed lambs after repeated infection with the GIN, *Teladorsagia circumcincta.* After comparing the parasitological data, histological techniques, immunohistochemistry, and ELISA studies were used to investigate the immune response at the mucosal level and transcriptomic analysis of the draining lymph node response to explore the local adaptive immune response.

## 2. Results

### 2.1. Parasitology

**Figure 1:**
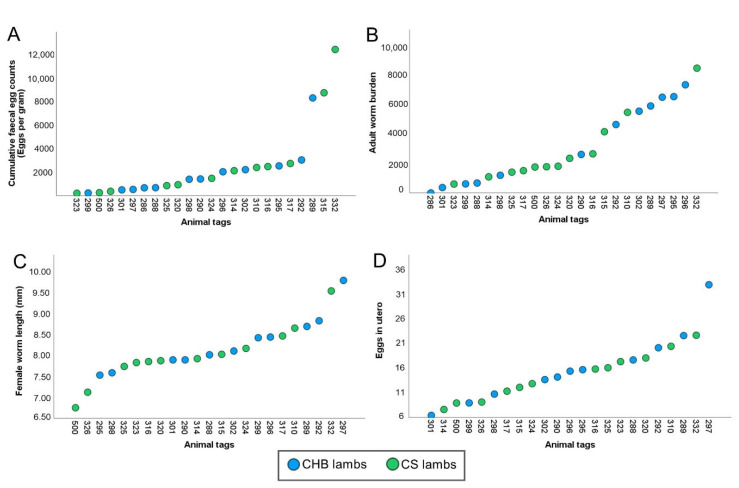
Parasitological variables in three-month-old sheep after trickle infection with *Teladorsagia circumcincta* infective larvae. Total cumulative faecal egg counts (**A**), worm burden at post-mortem (**B**), length of female worms (**C**), and eggs in utero (**D**) are shown as individual means. Animal tags in the X-axis are arranged from left to right according to ascending mean value (*n* = 24).

**Figure 2:**
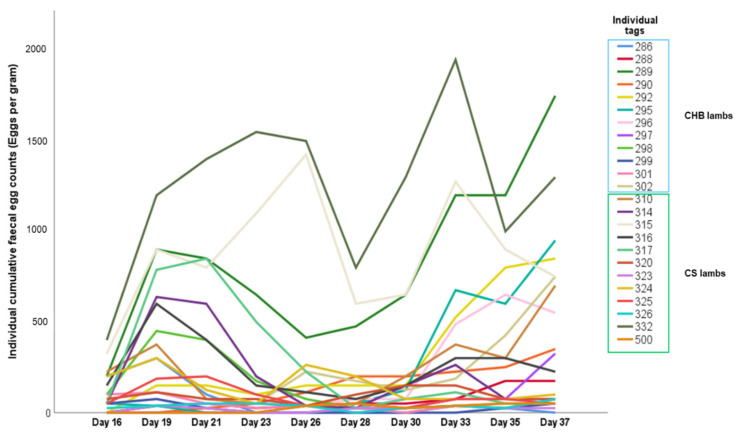
Individual cumulative faecal egg counts in three-month-old sheep after trickle infection with *Teladorsagia circumcincta* infective larvae (*n* = 24).

At the end of the trial, differences in cFEC, worm burden, female worm length, and eggs in utero were not statistically significant between breeds (Figure 1). Therefore, all animals were studied within the same group. Mean cFEC, adult worm burden, female worm length (mm), and eggs in utero values were 2429 (±623), 3128 (±518), 8.13 (±0.14), and 15 (±1), respectively. In addition, values showed high variability between individuals (Figure 1A–D). Individual results on faecal egg excretion throughout the experiment are represented in Figure 2 and Table 1. Nevertheless, several parasitological values were positively correlated, implying that those lambs with lower egg excretion also harboured fewer, shorter and less prolific parasites (Table 2).

### 2.2. Humoral and Cellular Immune Response

Levels of somatic L3, L4, and adult *T. circumcincta* antigen-specific IgA within the abomasal mucus, and several immune cell populations within the abomasal mucosa were quantified at post-mortem (Table 3). Correlation studies between antigen-specific IgA levels or abomasal cell populations with parasitological variables showed significant negative associations between several of these parameters (Table 3). Mucosal IgA targeting *T. circumcincta* L3, L4, and adult antigens was significantly negatively correlated with the number of eggs in utero. Additionally, mucosal IgA levels against the adult stage were significantly negatively associated with worm burden. Numbers of MCHII^+^ cells were significantly negatively associated with cFEC and worm burden. Lastly, globule leukocyte numbers were significantly negatively correlated with cFEC.

### 2.3. Transcriptomic Analysis

**Figure 3:**
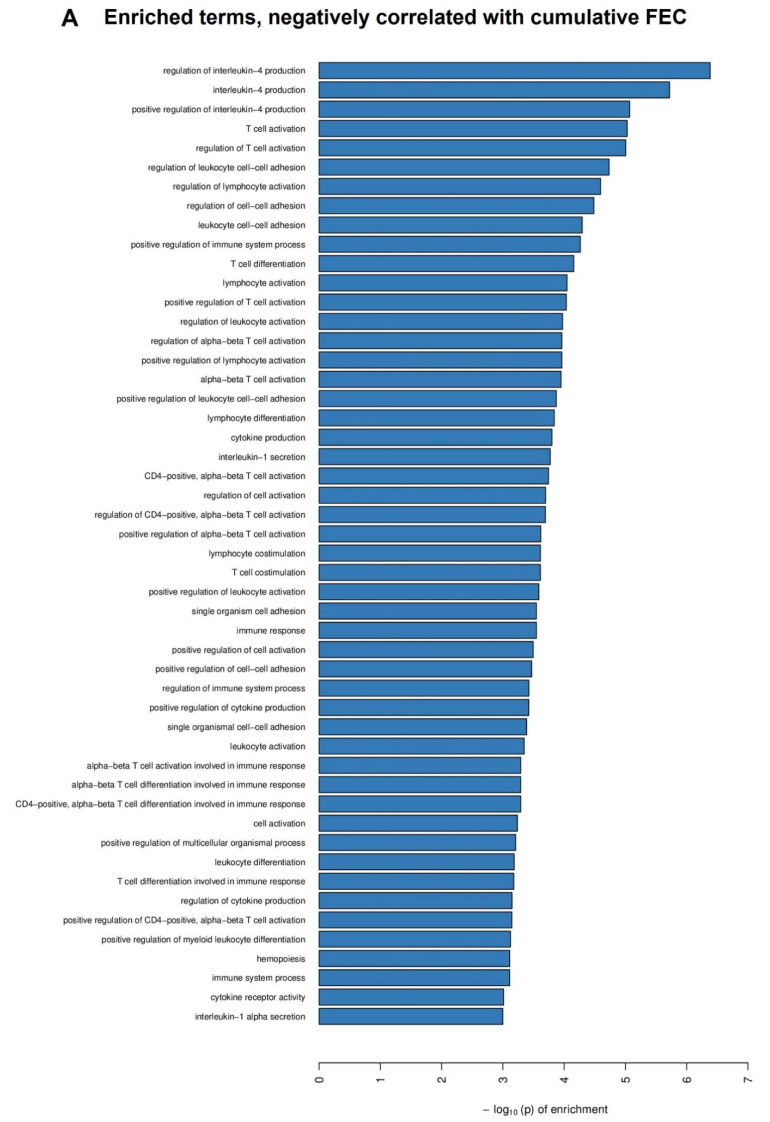

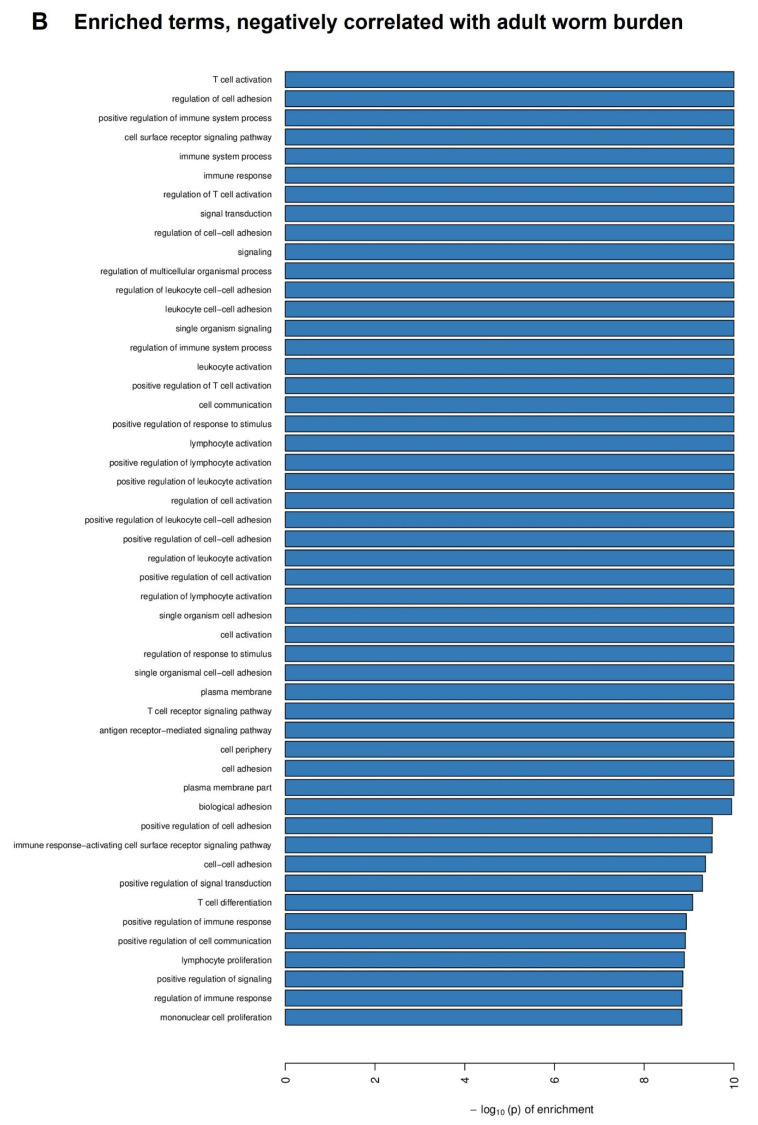
The top 50 enriched terms identified in a transcriptomic analysis of abomasal lymph nodes from three-month-old sheep after trickle infection with *Teladorsagia circumcincta* infective larvae. (**A**) depicts the transcripts negatively correlated with cumulative faecal egg counts and (**B**) depicts the transcripts negatively correlated with worm burden. Enrichment analyses with enrichment score are represented on the X axis and GO terms are displayed along the Y axis (*p* < 0.01).

The relationships between cFEC and worm burden with gene expression in the abomasal lymph node tissue were evaluated using linear regression models. When comparing the top 50 enriched terms for genes negatively associated across the cFEC and worm burden contrasts, 48% of terms overlapped and the majority were immunity and cell adhesion-related. For cFEC correlation analysis, expression of transcripts involved in IL-4 production, membrane, and signalling terms were enriched (Figure 3A). Similarly, for the observation of decreased worm burdens, transcripts involved in cell cycle and immune-related terms were enriched (Figure 3B). Thirty-eight and 148 significantly upregulated genes were involved in the top 50 pathways negatively correlated with cFEC and worm burden comparisons, respectively. Full details regarding individual significant genes included in each GO term for cFEC and worm burden contrasts can be found in Supplementary File S1 (Table S1A,B) and Supplementary File S2 (Table S2A,B), respectively.

## 3. Discussion

In contrast to previous experimental GIN infection studies that included older (approximately 6 month-old) animals [16,23], we found no evidence that CHB lambs were more resistant to *T. circumcincta* than CS lambs at three months of age. However, great individual variability in resistance regarding parasitological parameters was observed within both breeds. On this basis, the analysis here focused on characterising the humoral and cellular immune response among individuals in relation to their *T. circumcincta* burdens and faecal egg excretion profiles. The analysis indicated that most of the correlations between humoral and cellular variables and parasitological parameters were not significant. However, MHC-II+ cells, globule leukocytes and *T. circumcincta*-specific IgA appeared to have a role in controlling the infection with the parasite. By comparing transcriptional data from the abomasal lymph node with cFEC, worm burden and gene expression, negative correlations were identified between these parasitological parameters and multiple enriched immune-related GO terms and key immunological genes.

Gastrointestinal nematode infections in lambs represent one of the main concerns for the sheep industry; these parasites have a major negative impact on animal welfare and considerably contribute to poor production performance and economic loss. This is partly due to the relative immaturity of the lamb immune system during the first months of life [24,25], in which adaptive immune responses appear to be poor [26]. In addition, lambs generally require repeated contact with the parasites whilst grazing to develop a protective response against these nematodes [12]. A high individual variation in FEC excretion has been demonstrated in three-month-old lambs, implying that some animals can control GINs better than others at this age and prior data suggest that these differences are sustained over time [12]. This is similar to the unequal distribution of GIN within a normal population of adult sheep, where a small proportion of susceptible animals harbour most of the parasitic burden [27,28]. Hence, exploring the differences in immune response and dynamics in young animals could unravel the basis for resistance to these parasites from an early stage. 

In this regard, a recent comparative study showed that suckling Santa Ines lambs were more resistant than Ile de France lambs due to a strong abomasal cellular immune response when infected with *Haemonchus contortus,* with significant intra-breed variability [18]. The CHB and the CS breeds have been used previously as models in several studies because of differences in their resistance to *H. contortus* and *T. circumcincta* in lambs older than six months of age [16,23]. Moreover, a recent study demonstrated that three -month-old CHB lambs responded to vaccination with recombinant *T. circumcincta* proteins, with reductions in parasitological variables and involvement of well-characterised anti-GIN immune mechanisms described in adult animals, whereas such differences were not observed in three-month-old CS lambs [29]. In contrast, here, significant differences in susceptibility to *T. circumcincta* in unvaccinated lambs of these two breeds at three months of age were not identified. Interestingly, as alluded to above, great variability in individual response to infection was detected. Hence, data from both breeds was used in this study to examine the relationship between parasitological variables with the abomasal immune cell recruitment, local IgA production, and the analysis of the regional lymph node dynamics to explore possible resistance mechanisms. While this would not uncover breed-specific mechanisms of resistance to *T. circumcincta*, it allowed the investigation of resistance mechanisms in operation at a young age that are shared between the two breeds.

In general, the immune response correlated well with parasitological differences. Animals with lower worm and egg burdens had a higher expression of pathways related to inflammation cascades, such as cell migration and adhesion, hematopoiesis, mononuclear cell proliferation, response to stimulus, cell activation, and signalling (see Figure 3A,B). This is consistent with recent research on gene expression profiles in selected resistant and susceptible Yichang goats in which 31 genes related to immune processes and cell adhesion molecules pathways were more highly expressed in a more resistant group [30].

In addition, several pathways that were negatively associated with cFEC and worm burden contained genes involved in dendritic cell activation (*CCR7*, *FLT3*) and antigen presentation through MHC-I (*B2M*, *LMO7*) and MHC-II (*ARL14*) molecules. Counts of abomasal MHC-II^+^ cells were negatively associated with egg excretion and the abomasal worm population. These results are in line with the characteristics of anti-nematode adaptive immune responses, which usually start with macrophages or dendritic cells presenting parasitic antigens to naïve CD4^+^ T helper cells through MHC-II receptors, leading to their activation and differentiation towards a protective type 2 phenotype [11,31]. Similarly, a comparative study between GIN-resistant and susceptible Scottish Blackface lamb strains infected with *T. circumcincta* showed that resistant animals had a higher expression of pathways involved in inflammatory responses and attraction and binding of T-lymphocytes early in infection, implying they had quicker cell migration responses after challenge than susceptible lambs [32]. Early expression of immune processes and intermittent activation of type 2-related pathways involving CD4^+^ T cells and IL-4 expression in abomasal mucosa have also been reported in resistant Creole goat kids infected with *H. contortus* for 35 days [21]. Amongst the top 50 pathways negatively associated with cFEC and worm burden were the regulation and differentiation of leukocytes, lymphocytes, T cells, γδ T cells and CD4^+^ T cells and IL-4 cytokine production. These pathways reflect a classical type 2 response, likely leading to increases in IL-4, IL-13, and IL-5 production, prompting IgE and IgA antigen-specific production by B cells, along with recruitment of mast cells, globule leukocytes, and eosinophils [31,32]. In this study, some genes were implicated in B cell activation and immunoglobulin production, related to the regulation of the IgE receptor (*DOCK10*). Globule leukocytes, thought to be degranulated mast cells, have been linked with the expulsion of worms from their niche through contraction of the abomasal smooth muscle and the stimulation of mucus secretion [10,33]. Interestingly, globule leukocytes were negatively correlated with egg excretion here but not with the abomasal worm population. In addition, mast cells and globule leukocyte degranulation is associated with IgE production [34]. Several genes related to mast cell activation and degranulation (*EDN1*, *PTGDR*, *RASGRP1*) were present in several pathways negatively associated with parasitological parameters. Moreover, levels of antigen-specific IgA were correlated with reduced egg production in utero and worm establishment here, which is in line with the literature, which associates the presence of this immunoglobulin isotype with impaired nematode development and detrimental effects on worm length and fecundity [35].

In conclusion, the lambs that controlled parasitological parameters more efficiently developed protective type-2 immune responses, similar to those developed by adult animals in other studies [23]. Future study designs will confirm the data presented; for example, by creating resistant and susceptible lineages and exploring the dynamics of the immune response with changes in gene expression throughout infection. This strategy will be key to identifying immune responses associated with protection, thus pinpointing resistance genetic markers for the selection of animals for breeding.

## 4. Materials and Methods

### 4.1. Animals and Parasitology

The experimental design was previously described in detail in the study of Pérez-Hernández et al. [29]. Briefly, three-month-old CHB (*n* = 12) and CS (*n* = 12) male lambs were purchased from several farms in Gran Canaria and were dewormed on arrival with fenbendazole. Coprological examination was performed two weeks after drenching to confirm the effectiveness of the anthelmintic treatment and animals were kept in conditions that excluded further parasite transmission until the start of the trial. All animals were challenged with 2000 *T. circumcincta* L3 (MTci2 strain, Weybridge, UK) three times a week for a total of four weeks (from day 0 to day 26). 

Starting on day 14, faeces were directly collected from the rectum of the lambs three times per week until day 37 after the start of the infection regime. Faecal egg counts (FEC, measured as eggs per gram, EPG) were determined using the modified McMaster technique (sensitivity 50 EPG). At the end of the experiment (days 40–43), animals were euthanised to recover juvenile and adult parasites from aliquots of the abomasal contents. Estimations of worm burden, female worm length, and numbers of eggs in utero were performed following the previously described protocols [16,36].

### 4.2. Enzyme-Linked Immunological Assay (ELISA)

The ELISA protocol was previously described in Pérez-Hernández et al. [29]. Mucus samples were obtained at post-mortem (days 40–43) from the abomasal surface after washing and preserved at −80 °C until use. ELISA was used to assess mucosal IgA levels against native *T. circumcincta* antigens (somatic L3, L4, and adult, 5 µg/mL plate coating concentration). Each sample was assayed in duplicate, and all test plates contained positive and negative control samples to account for plate-to-plate variation. The optical densities (OD) were transformed into an optical density index (ODI), using the formula: ODI = (mean OD − mean negative control OD)/(mean positive control OD − mean negative control OD), as previously described by Strain and Stear, [37]. These values were then increased by a value of 1.0 to avoid negative values and to prevent statistical errors [23,37].

### 4.3. Histology and Immunohistochemistry

Full details of this protocol are published in Pérez-Hernández et al. [29]. Briefly, at post mortem, two abomasal tissue samples of the antropyloric region were taken. One sample was processed and embedded in paraffin-wax and stained with hematoxylin and eosin to count eosinophil and globule leukocyte numbers, whilst toluidine blue staining was used to determine mast cell numbers. Cells were counted adjacent to the lamina propria (eosinophils and mast cells) or in the luminal margin of the mucosa (globule leukocytes) and expressed as cells/mm^2^. 

The other tissue sample was embedded in OCT^TM^ solution (Optimal Cutting Temperature, Tissue Tek, Sakura Finetek Europe B.V., Zoeterwoude, The Netherlands), preserved at −80 °C and sectioned with a cryostat. Immunohistochemical staining was performed as described previously by González et al. [38]. Primary antibodies against CD4, CD8, γδ, MHC-II, and Galectin 14 sheep markers were used to stain the tissue sections. Positively stained cells were counted in the upper and lower half of the mucosa [29]. 

### 4.4. RNA Extraction and Sequencing

Abomasal lymph node samples were collected at post-mortem and kept in E.Z.N.A. RNA Lock Reagent (Omega Bio-tek Inc, Norcross, GA, USA) at −80 °C for RNA extraction. Total RNA was extracted from the lymph node samples by first cutting the primary sample (20 mg) into smaller pieces with a sterile scalpel and then further homogenising each sample in 1 mL RLT buffer (Qiagen Ltd., Manchester, UK) using a Precellys bead basher (Bertin Instruments, Basingstoke, UK) within a Precellys CK28 bead tube (Stretton Scientific, Stretton, UK) for 3 × 23-s cycles at 5800 rpm with 2 min between cycles, on ice. Samples were cleared by centrifugation at 14,000× *g* at 4 °C for 10 min and supernatants processed using a RNeasy mini-isolation kit (Qiagen Ltd., Manchester, UK) according to the manufacturer’s protocol and including an on-column DNase I digestion for 15 min at room temperature. RNA quantity and integrity were assessed using a Nanodrop spectrophotometer (Thermo Fisher, Inchinnan, UK) and a Bioanalyser RNA Nanochip (Agilent Technologies Ltd., Cheadle, UK). The mean RNA integrity number (RIN) value across all samples was 8.1, indicating the successful extraction of high-quality RNA from the abomasal lymph nodes. Prior to sequencing library preparation, the yield of total RNA was determined on a Qubit Fluorometer (ThermoFisher, Inchinnan, UK) using the Broad Range RNA kit (Thermo Fisher, Inchinnan, UK). The resulting RNA samples were sequenced on an Illumina HiSeq 4000 by The Centre for Genome Research (CGR) at the University of Liverpool, Liverpoool, UK, generating 2 × 150 bp strand-specific, paired-end reads with a minimum of 30 million reads per sample.

### 4.5. RNA-Seq Quality Control and Alignment

R version 3.4.4 (2018-03-15) was used for the RNAseq data QC based on all 21 samples with the array QualityMetrics package. Base calls were made using the Illumina CASAVA 1.8 pipeline and cutadapt (v1.11) was used for adapter trimming. Processed sequences were then aligned to the *Ovis aries* genome assembly Oar_v3.1 (GCA_000298735.1) using the STAR aligner and the numbers of mapped read pairs were counted based on the *O. aries* genome annotation (Ensembl release 91) also within STAR [39].

### 4.6. Statistical Analysis

Statistical analysis and graph production were conducted using the IBM SPSS Statistics 24.0 programme. The total number of eggs excreted during the experiment (cumulative FEC or cFEC) were estimated using the trapezoidal method for calculation of area under the curve [16]. Parasitological data were initially compared between breeds and evaluated using a Generalised Linear Model [23]. Thereafter, animals from both breeds were considered together as a single group. Associations between parasitological parameters, immunoglobulin level data, and mucosal cellular counts were evaluated through Spearman’s correlation coefficients. Probabilities with *p*-value < 0.05 were considered statistically significant. Two linear regression models were performed, examining the relationships between levels of gene expression and cFEC or worm burden. Functional enrichment analysis was performed to identify gene ontology (GO) terms that were enriched in genes with statistically significant associations with parasitological parameters using GO annotations obtained from Ensembl. Significantly associated genes (raw *p*-value < 0.05, up to a maximum of 1000 genes ranked by *p*-value) from each comparison were analysed for enrichment of GO terms across all three GO categories using a hypergeometric test; this was then corrected for with tests over multiple terms using a Benjamini-Hochberg correction (FDR correction) to yield an adjusted *p*-value (*p* < 0.01).

## Figures and Tables

**Table 1:** Individual cumulative faecal egg counts in three-month-old sheep after trickle infection with *Teladorsagia circumcincta* infective larvae (*n* = 24).

Breed	Individual Tag	Day 16	Day 19	Day 21	Day 23	Day 26	Day 28	Day 30	Day 33	Day 35	Day 37
CHB	286	200	300	100	0	0	0	0	38	25	0
	288	0	38	50	25	38	50	50	75	175	175
	289	200	900	850	650	413	475	650	1200	1200	1750
	290	0	0	25	50	113	200	200	225	250	350
	292	0	150	150	100	150	150	150	525	800	850
	295	50	38	0	0	38	50	125	675	600	950
	296	50	75	25	0	38	75	75	488	650	550
	297	0	0	0	0	0	25	25	75	75	325
	298	100	450	400	175	75	25	25	38	25	75
	299	50	75	25	0	0	0	0	0	25	50
	301	100	113	50	25	38	25	0	38	50	50
	302	75	113	75	50	225	175	125	188	425	750
CS	310	225	375	75	50	38	50	200	375	300	700
	314	50	638	600	200	38	25	150	263	75	75
	315	325	900	800	1100	1425	600	650	1275	900	750
	316	150	600	400	150	113	75	150	300	300	225
	317	50	788	850	500	225	25	75	113	50	50
	320	75	113	75	75	38	100	150	150	75	75
	323	0	38	25	0	0	25	25	38	25	25
	324	200	300	125	50	263	200	75	75	75	100
	325	50	188	200	100	38	50	25	75	75	50
	326	25	38	50	50	38	0	25	38	25	75
	332	400	1200	1400	1550	1500	800	1300	1950	1000	1300
	500	0	0	0	0	38	50	25	38	50	50

**Table 2:** Parasitological correlations in three-month-old sheep after trickle infection with *Teladorsagia circumcincta* L3.

	Adult Worm Burden	Female Worm Length	Eggs in Utero
Cumulative FEC	0.654 **	0.476 *	0.332
Adult worm burden	-	0.464 *	0.578 **
Female worm length		-	0.501 *

Significant correlations between variables are indicated by * *p* < 0.05 and ** *p* < 0.01 (*n* = 24).

**Table 3:** Immunological means and correlations in three-month-old sheep after trickle infection with *Teladorsagia circumcincta* L3. Values are shown as mean Optical Density Index (ODI) for IgA expression or cells/mm^2^ for cell recruitment (*n* = 24).

		Correlation			
Variable	Mean (ODI or cells/mm^2^) ± SEM	Cumulative FEC	Adult Worm Burden	Female Worm Length	Eggs in Utero
Mucus IgA-L3	1.10 ± 0.07	−0.069	−0.241	−0.331	−0.669 **
Mucus IgA-L4	1.15 ± 0.04	−0.189	−0.294	−0.377	−0.668 **
Mucus IgA-AD	1.01 ± 0.02	−0.141	−0.543 **	−0.175	−0.419 *
CD4^+^	41.01 ± 7.45	−0.147	0.042	−0.209	0.035
CD8^+^	74.21 ± 16.59	−0.001	−0.198	0.045	0.082
γδ^+^	18.05 ± 2.93	−0.122	−0.065	−0.214	−0.126
MHC-II^+^	33.21 ± 8.50	−0.476 *	−0.476 *	−0.030	−0.269
Gal 14^+^	4.00 ± 0.88	−0.207	−0.104	−0.179	−0.180
Eosinophils	86.56 ± 10.73	−0.336	−0.375	0.126	−0.314
Globule leukocytes	172.70 ± 41.13	−0.443 *	−0.350	−0.287	−0.323
Mast cells	29.93 ± 6.91	−0.129	−0.137	−0.159	−0.251

Significant correlations between variables are indicated by * *p* < 0.05 and ** *p* < 0.01. L3: *T. circumcincta* third-stage larvae; L4: *T. circumcincta* fourth-stage larvae; AD: *T. circumcincta* adult stage.

## Data Availability

The data obtained from this study are available from the corresponding author upon reasonable request.

## Supplementary Materials

The following supporting information can be downloaded at: https://www.mdpi.com/article/10.3390/ijms24010029/s1.
